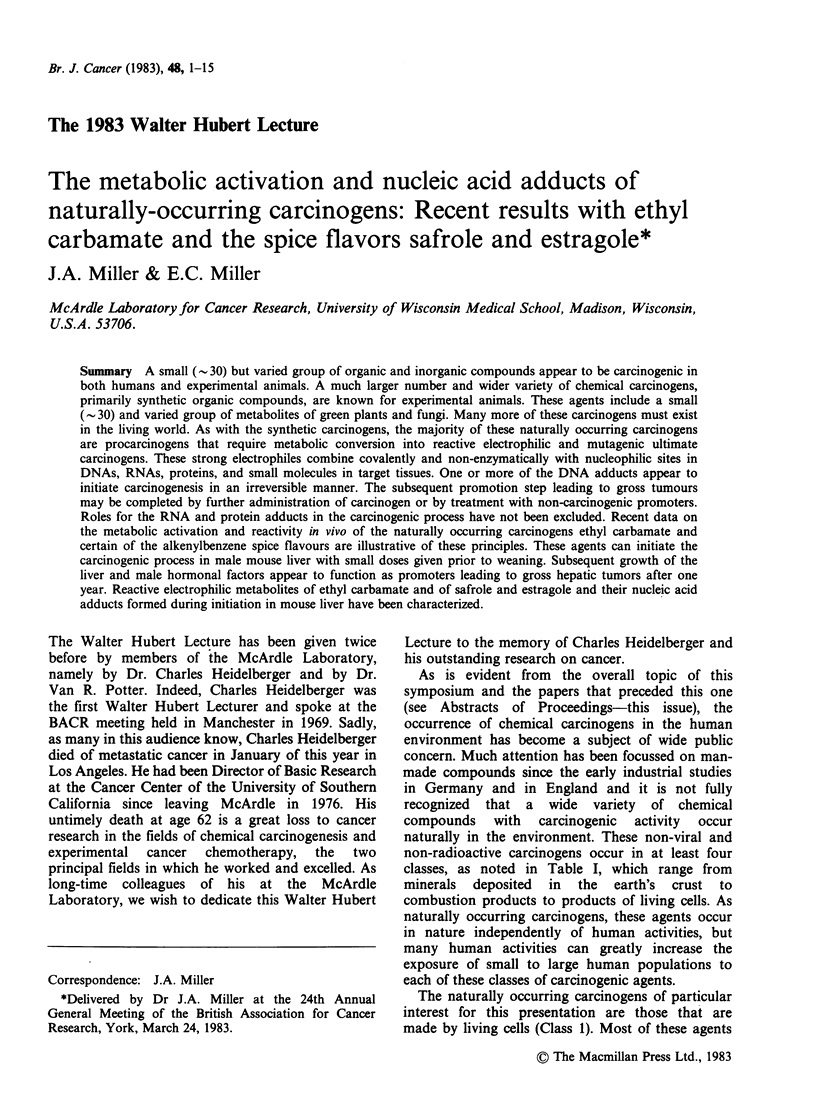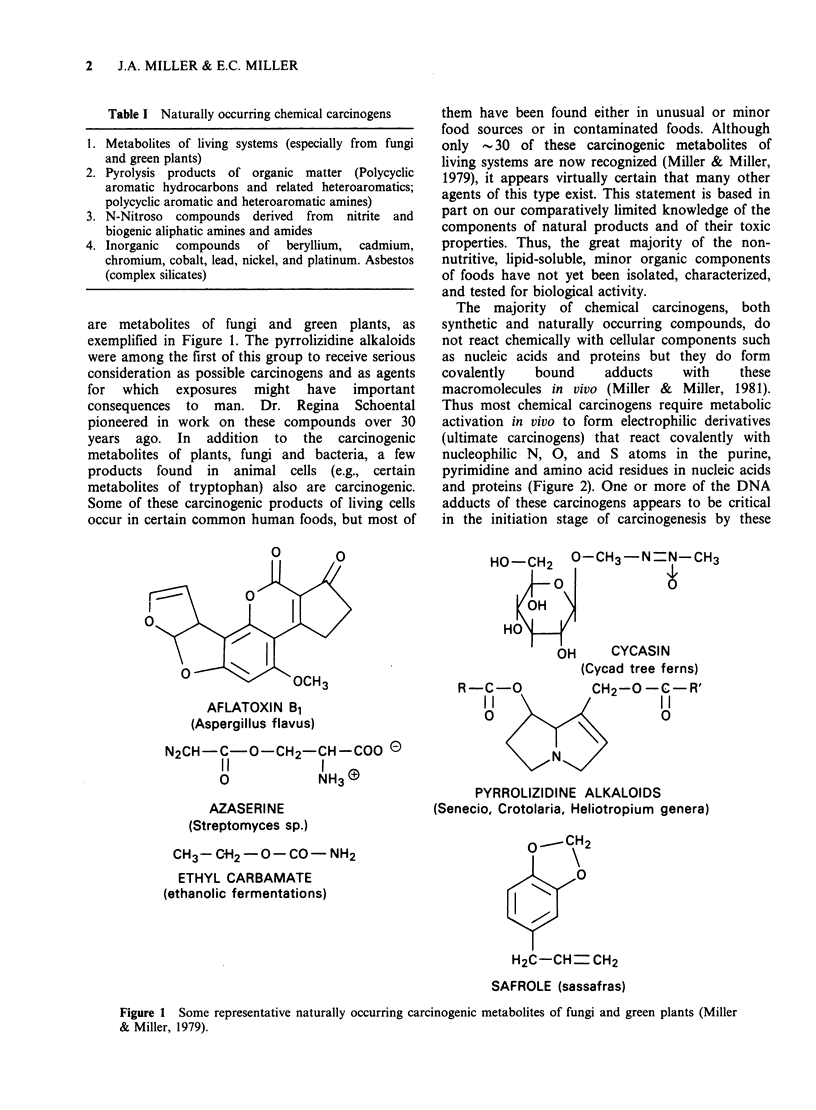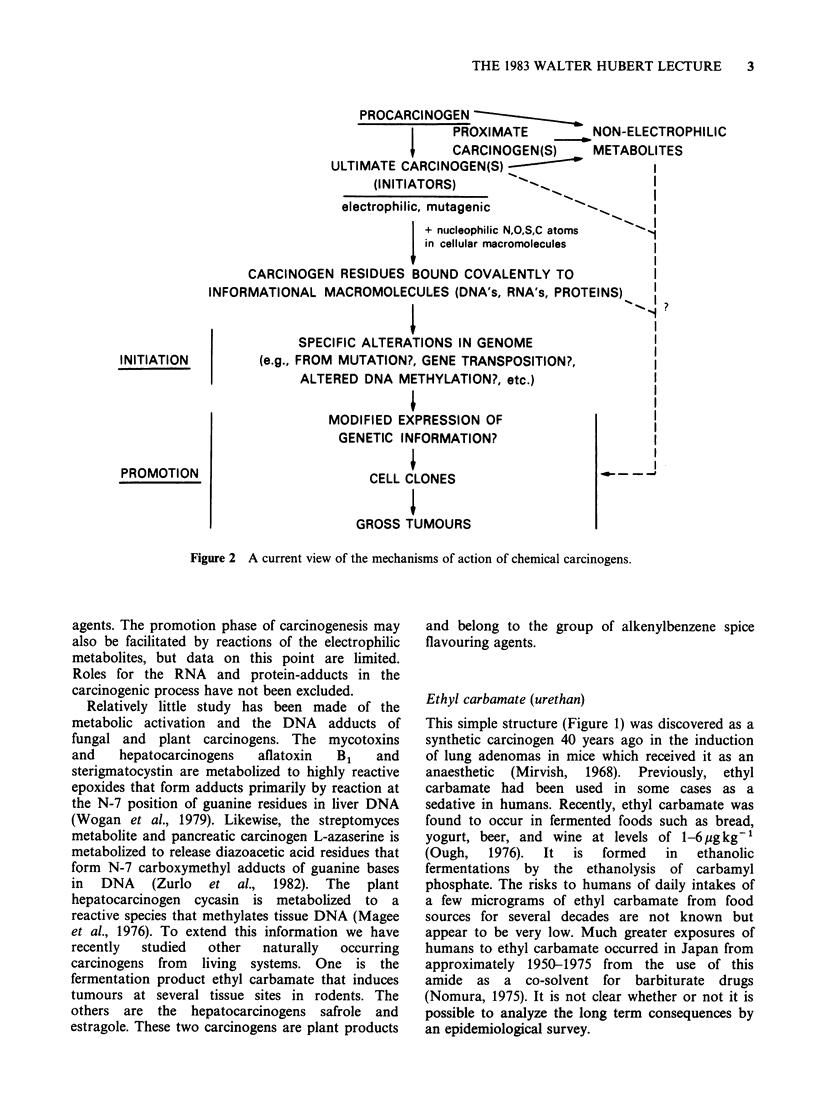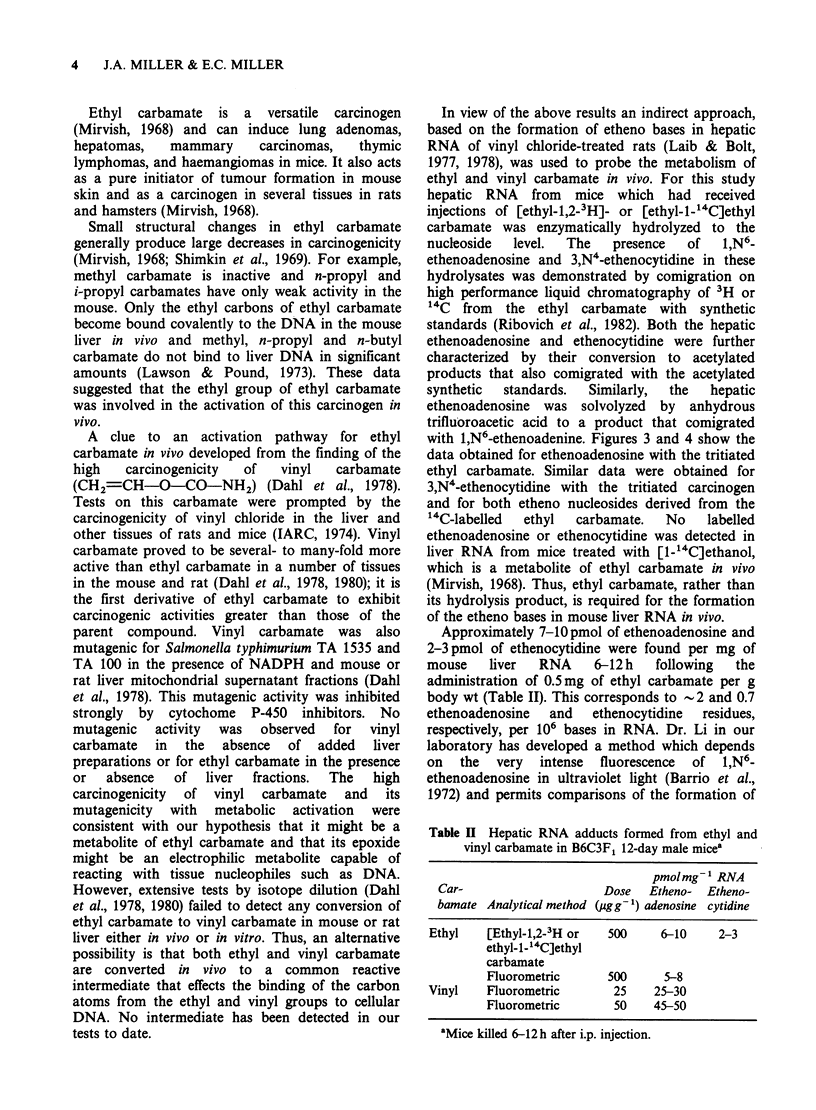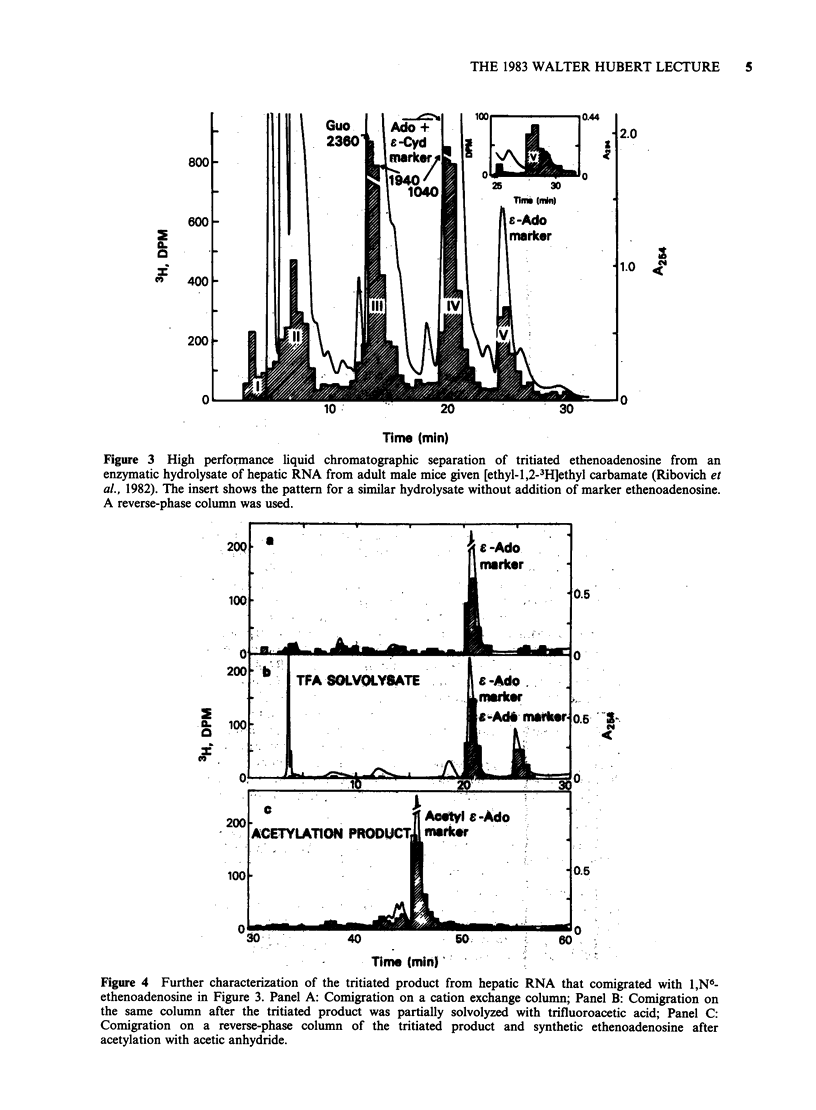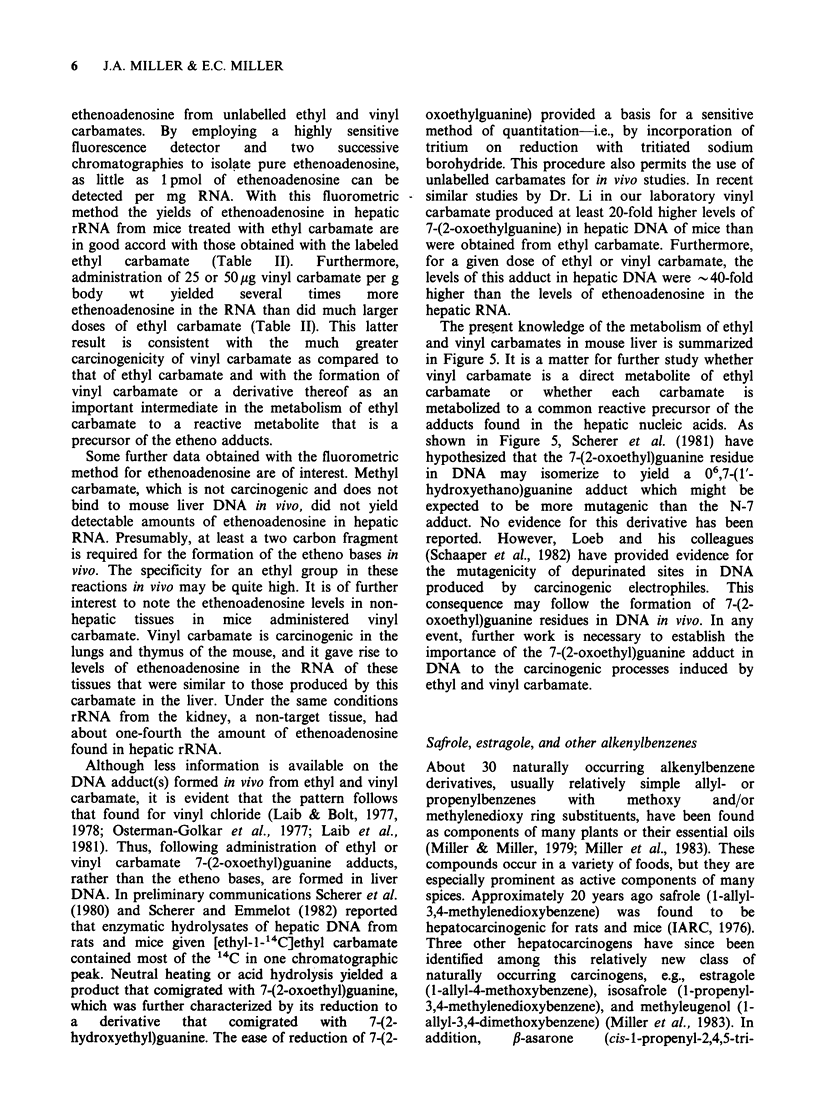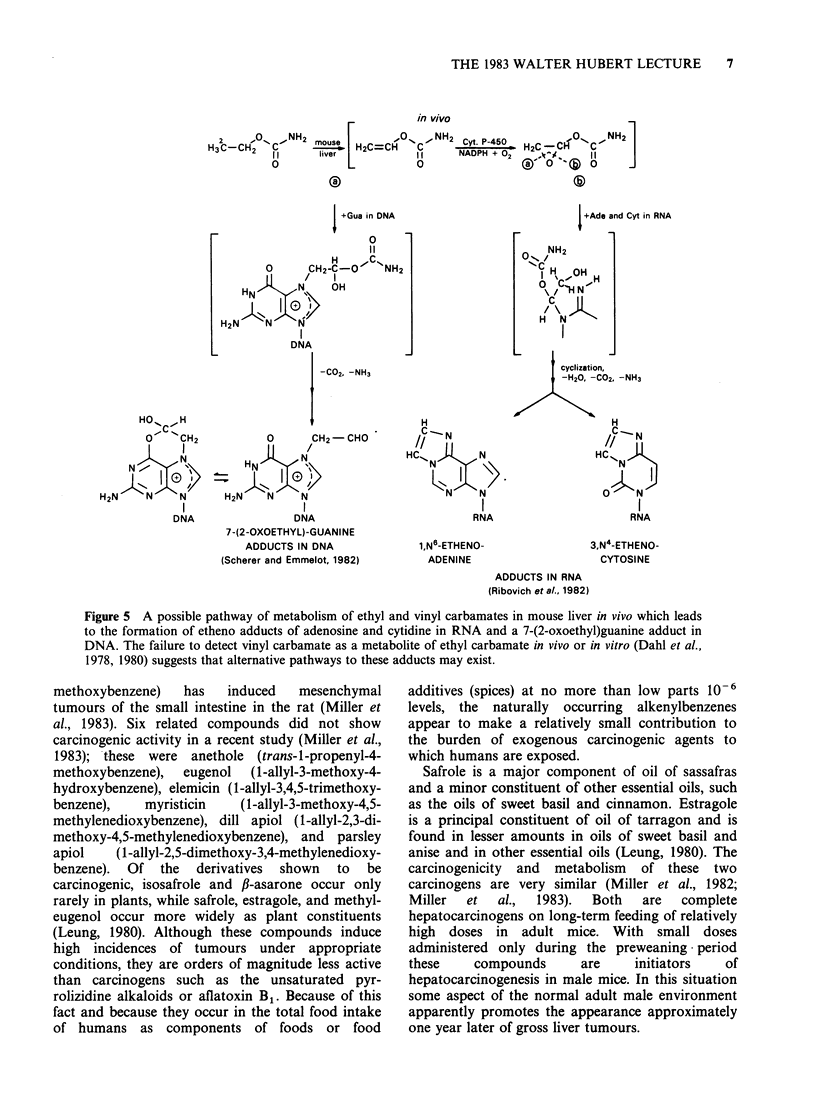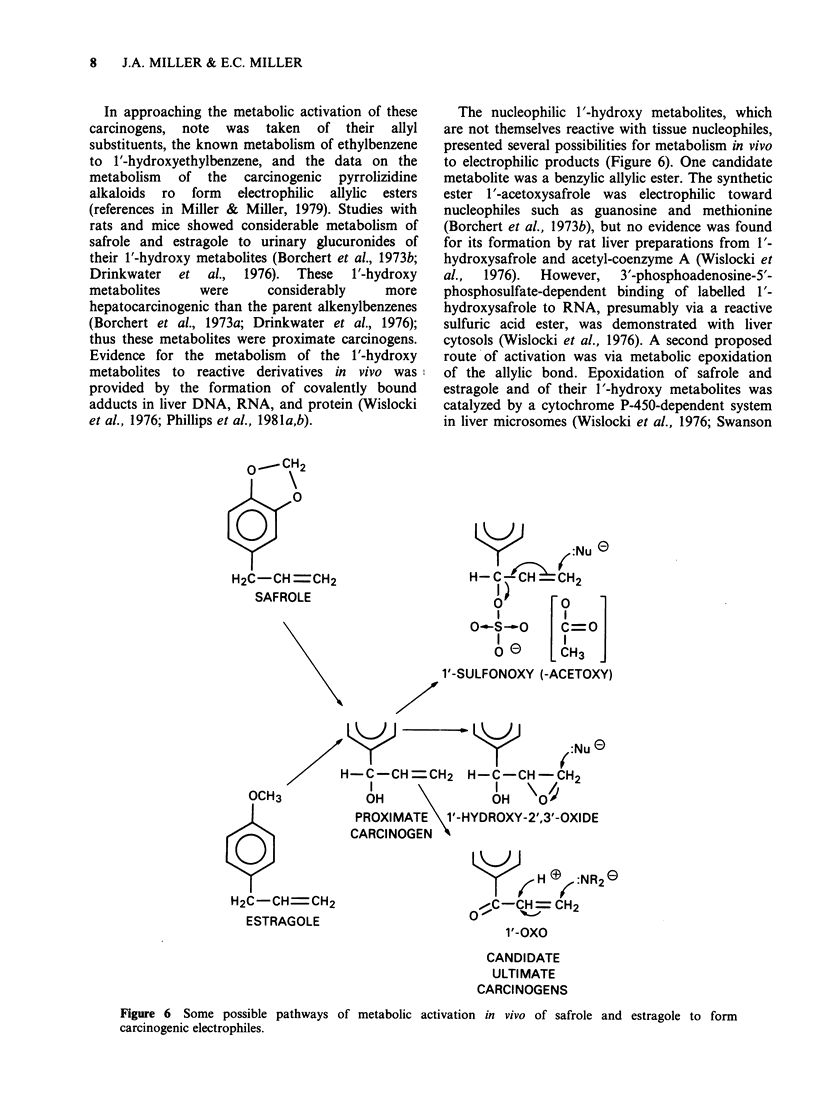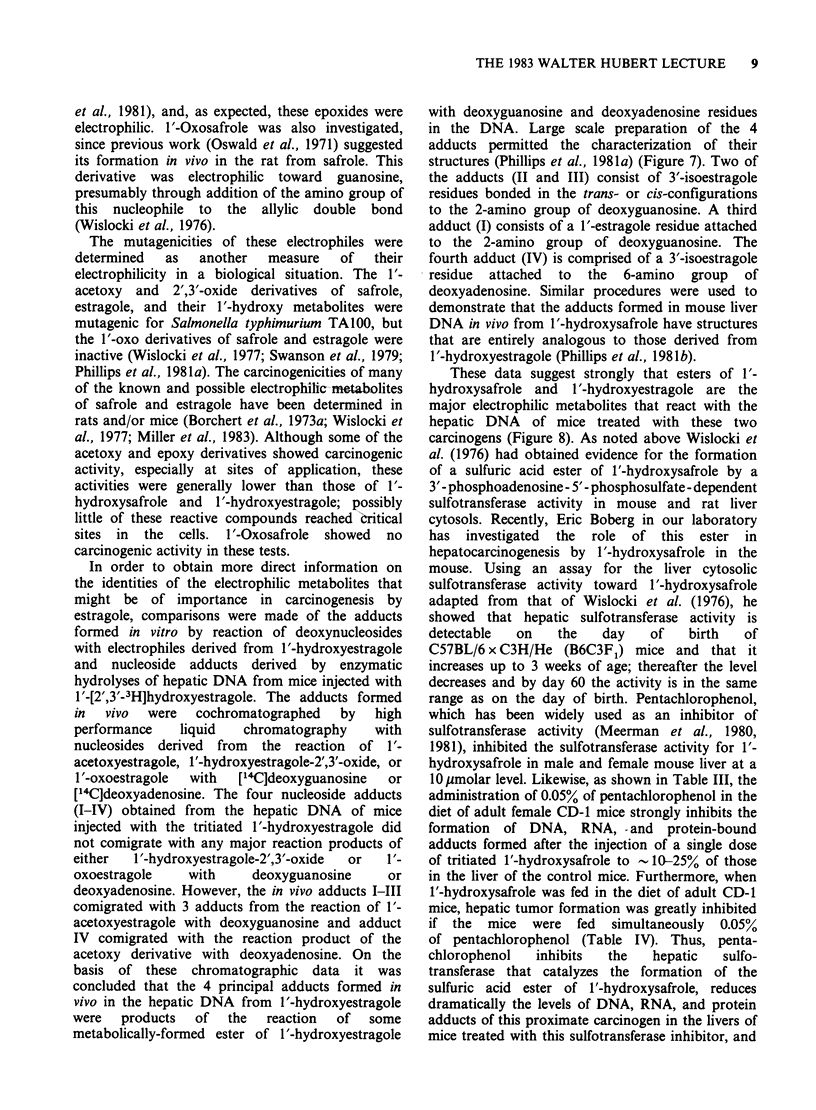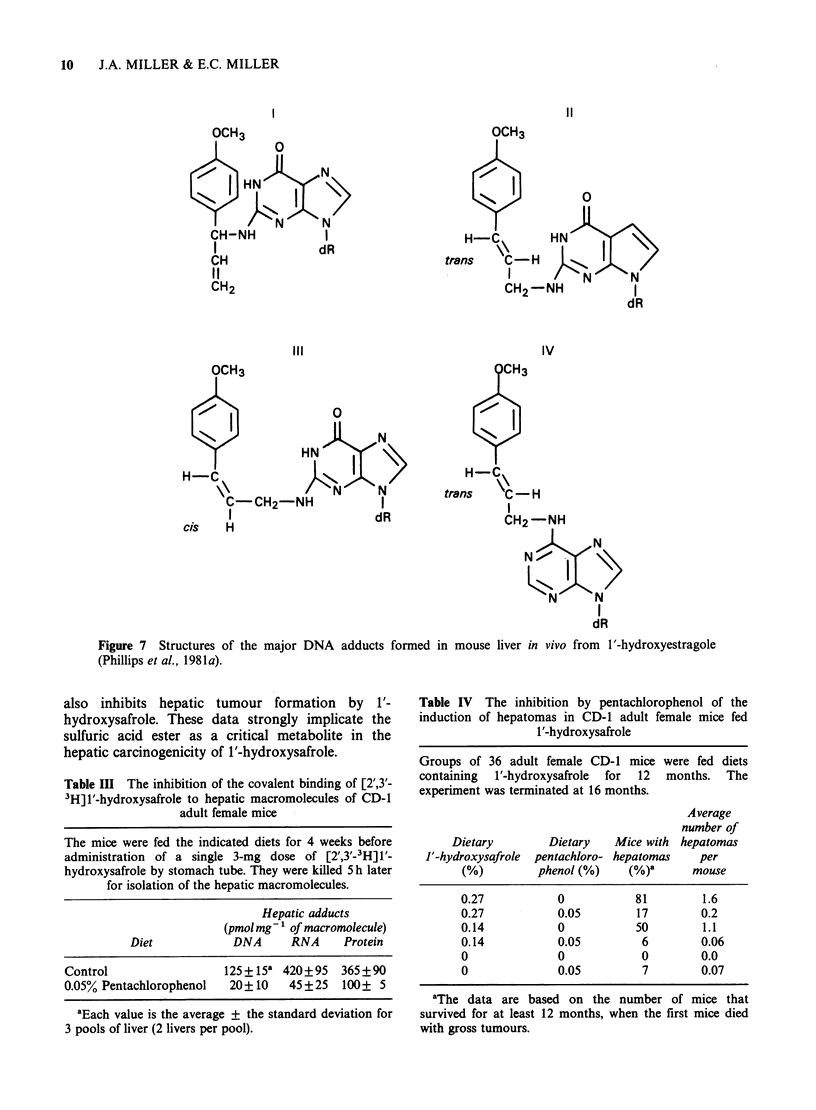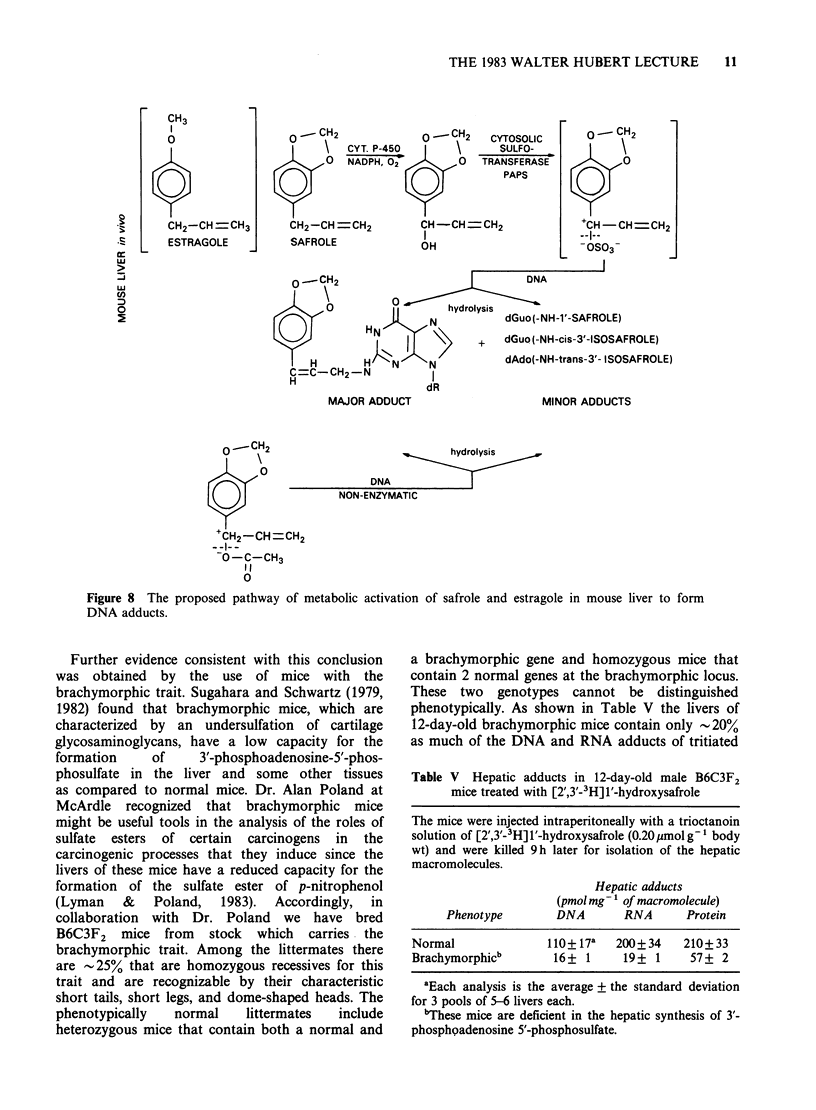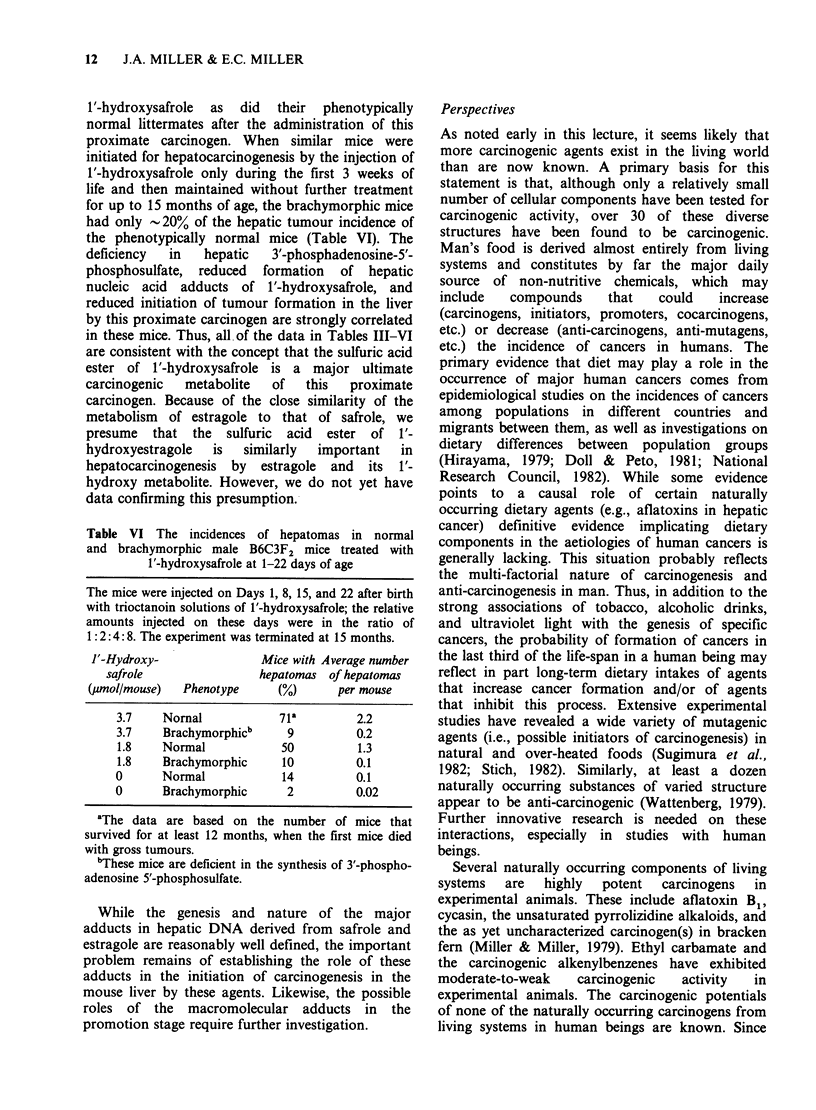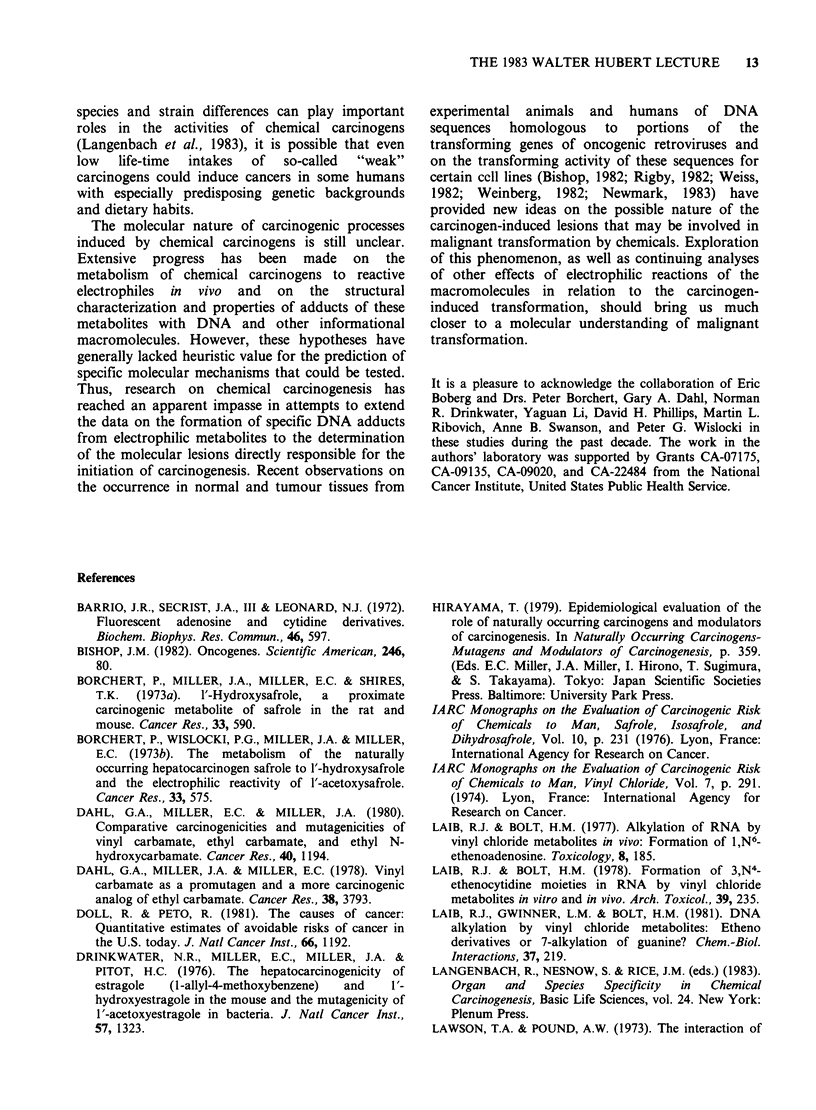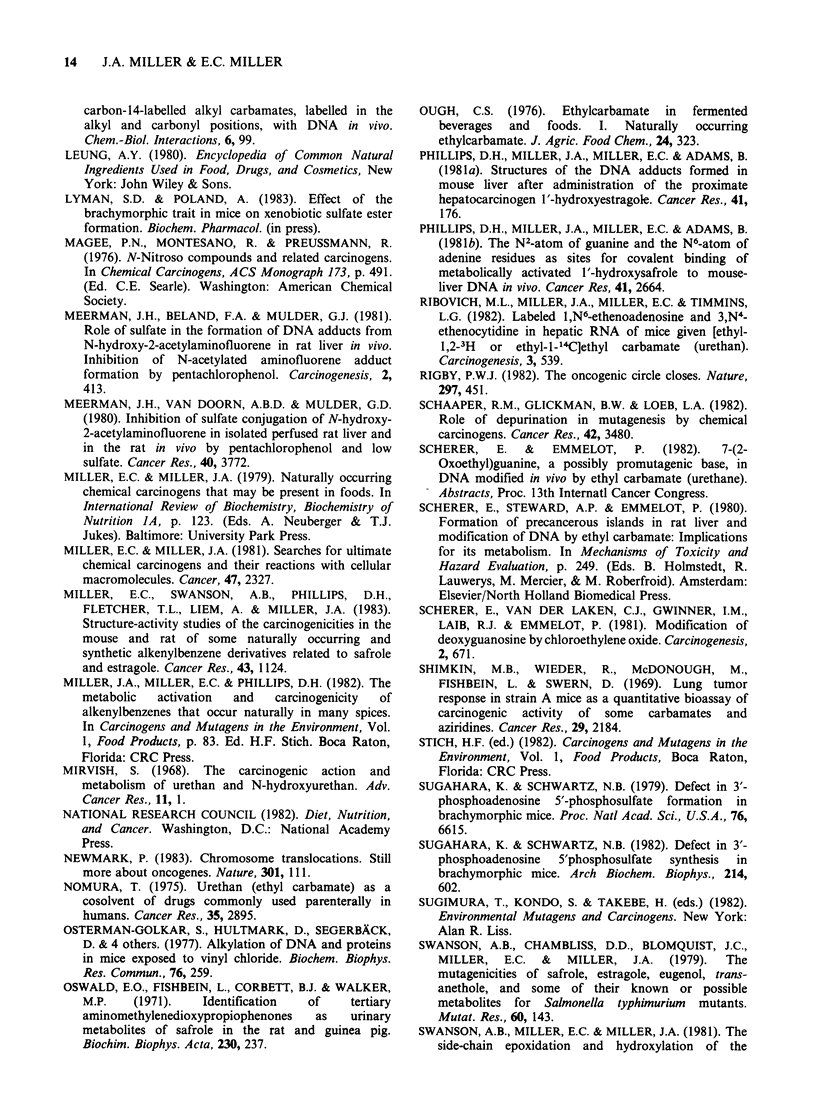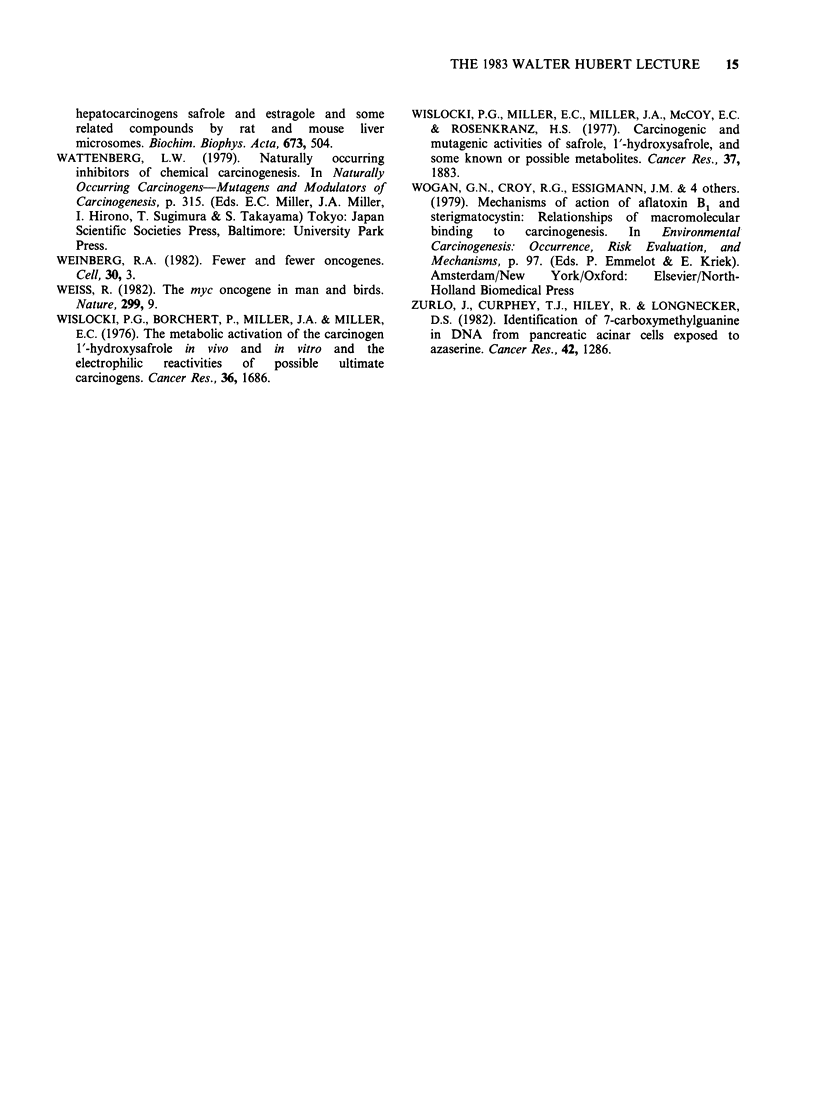# The metabolic activation and nucleic acid adducts of naturally-occurring carcinogens: recent results with ethyl carbamate and the spice flavors safrole and estragole.

**DOI:** 10.1038/bjc.1983.151

**Published:** 1983-07

**Authors:** J. A. Miller, E. C. Miller

## Abstract

A small (approximately 30) but varied group of organic and inorganic compounds appear to be carcinogenic in both humans and experimental animals. A much larger number and wider variety of chemical carcinogens, primarily synthetic organic compounds, are known for experimental animals. These agents include a small (approximately 30) and varied group of metabolites of green plants and fungi. Many more of these carcinogens must exist in the living world. As with the synthetic carcinogens, the majority of these naturally occurring carcinogens are procarcinogens that require metabolic conversion into reactive electrophilic and mutagenic ultimate carcinogens. These strong electrophiles combine covalently and non-enzymatically with nucleophilic sites in DNAs, RNAs, proteins, and small molecules in target tissues. One or more of the DNA adducts appear to initiate carcinogenesis in an irreversible manner. The subsequent promotion step leading to gross tumours may be completed by further administration of carcinogen or by treatment with non-carcinogenic promoters. Roles for the RNA and protein adducts in the carcinogenic process have not been excluded. Recent data on the metabolic activation and reactivity in vivo of the naturally occurring carcinogens ethyl carbamate and certain of the alkenylbenzene spice flavours are illustrative of these principles. These agents can initiate the carcinogenic process in male mouse liver with small doses given prior to weaning. Subsequent growth of the liver and male hormonal factors appear to function as promoters leading to gross hepatic tumors after one year. Reactive electrophilic metabolites of ethyl carbamate and of safrole and estragole and their nucleic acid adducts formed during initiation in mouse liver have been characterized.


Br. J. Cancer (1983), 48, 1–15

### The 1983 Walter Hubert Lecture

### The metabolic activation and nucleic acid adducts of naturally-occurring carcinogens: Recent results with ethyl carbamate and the spice flavors safrole and estragole*

J.A. Miller & E.C. Miller

*McArdle Laboratory for Cancer Research, University of Wisconsin Medical School, Madison, Wisconsin, U.S.A. 53706.*

**Summary** A small (~30) but varied group of organic and inorganic compounds appear to be carcinogenic in both humans and experimental animals. A much larger number and wider variety of chemical carcinogens, primarily synthetic organic compounds, are known for experimental animals. These agents include a small (~30) and varied group of metabolites of green plants and fungi. Many more of these carcinogens must exist in the living world. As with the synthetic carcinogens, the majority of these naturally occurring carcinogens are procarcinogens that require metabolic conversion into reactive electrophilic and mutagenic ultimate carcinogens. These strong electrophiles combine covalently and non-enzymatically with nucleophilic sites in DNAs, RNAs, proteins, and small molecules in target tissues. One or more of the DNA adducts appear to initiate carcinogenesis in an irreversible manner. The subsequent promotion step leading to gross tumours may be completed by further administration of carcinogen or by treatment with non-carcinogenic promoters. Roles for the RNA and protein adducts in the carcinogenic process have not been excluded. Recent data on the metabolic activation and reactivity *in vivo* of the naturally occurring carcinogens ethyl carbamate and certain of the alkenylbenzene spice flavours are illustrative of these principles. These agents can initiate the carcinogenic process in male mouse liver with small doses given prior to weaning. Subsequent growth of the liver and male hormonal factors appear to function as promoters leading to gross hepatic tumors after one year. Reactive electrophilic metabolites of ethyl carbamate and of safrole and estragole and their nucleic acid adducts formed during initiation in mouse liver have been characterized.

The Walter Hubert Lecture has been given twice before by members of the McArdle Laboratory, namely by Dr. Charles Heidelberger and by Dr. Van R. Potter. Indeed, Charles Heidelberger was the first Walter Hubert Lecturer and spoke at the BACR meeting held in Manchester in 1969. Sadly, as many in this audience know, Charles Heidelberger died of metastatic cancer in January of this year in Los Angeles. He had been Director of Basic Research at the Cancer Center of the University of Southern California since leaving McArdle in 1976. His untimely death at age 62 is a great loss to cancer research in the fields of chemical carcinogenesis and experimental cancer chemotherapy, the two principal fields in which he worked and excelled. As long-time colleagues of his at the McArdle Laboratory, we wish to dedicate this Walter Hubert Lecture to the memory of Charles Heidelberger and his outstanding research on cancer.

As is evident from the overall topic of this symposium and the papers that preceded this one (see Abstracts of Proceedings—this issue), the occurrence of chemical carcinogens in the human environment has become a subject of wide public concern. Much attention has been focussed on man-made compounds since the early industrial studies in Germany and in England and it is not fully recognized that a wide variety of chemical compounds with carcinogenic activity occur naturally in the environment. These non-viral and non-radioactive carcinogens occur in at least four classes, as noted in Table I, which range from minerals deposited in the earth's crust to combustion products to products of living cells. As naturally occurring carcinogens, these agents occur in nature independently of human activities, but many human activities can greatly increase the exposure of small to large human populations to each of these classes of carcinogenic agents.

The naturally occurring carcinogens of particular interest for this presentation are those that are made by living cells (Class 1). Most of these agents

Correspondence: J.A. Miller

*Delivered by Dr J.A. Miller at the 24th Annual General Meeting of the British Association for Cancer Research, York, March 24, 1983.

© The Macmillan Press Ltd., 1983

**Table I** Naturally occurring chemical carcinogens

1. Metabolites of living systems (especially from fungi and green plants)
2. Pyrolysis products of organic matter (Polycyclic aromatic hydrocarbons and related heteroaromatics; polycyclic aromatic and heteroaromatic amines)
3. N-Nitroso compounds derived from nitrite and biogenic aliphatic amines and amides
4. Inorganic compounds of beryllium, cadmium, chromium, cobalt, lead, nickel, and platinum. Asbestos (complex silicates)

are metabolites of fungi and green plants, as exemplified in Figure 1. The pyrrolizidine alkaloids were among the first of this group to receive serious consideration as possible carcinogens and as agents for which exposures might have important consequences to man. Dr. Regina Schoental pioneered in work on these compounds over 30 years ago. In addition to the carcinogenic metabolites of plants, fungi and bacteria, a few products found in animal cells (e.g., certain metabolites of tryptophan) also are carcinogenic. Some of these carcinogenic products of living cells occur in certain common human foods, but most of them have been found either in unusual or minor food sources or in contaminated foods. Although only ~30 of these carcinogenic metabolites of living systems are now recognized (Miller & Miller, 1979), it appears virtually certain that many other agents of this type exist. This statement is based in part on our comparatively limited knowledge of the components of natural products and of their toxic properties. Thus, the great majority of the non-nutritive, lipid-soluble, minor organic components of foods have not yet been isolated, characterized, and tested for biological activity.

The majority of chemical carcinogens, both synthetic and naturally occurring compounds, do not react chemically with cellular components such as nucleic acids and proteins but they do form covalently bound adducts with these macromolecules *in vivo* (Miller & Miller, 1981). Thus most chemical carcinogens require metabolic activation *in vivo* to form electrophilic derivatives (ultimate carcinogens) that react covalently with nucleophilic N, O, and S atoms in the purine, pyrimidine and amino acid residues in nucleic acids and proteins (Figure 2). One or more of the DNA adducts of these carcinogens appears to be critical in the initiation stage of carcinogenesis by these

**Figure 1** Some representative naturally occurring carcinogenic metabolites of fungi and green plants (Miller & Miller, 1979).

**Figure 2** A current view of the mechanisms of action of chemical carcinogens.

agents. The promotion phase of carcinogenesis may also be facilitated by reactions of the electrophilic metabolites, but data on this point are limited. Roles for the RNA and protein-adducts in the carcinogenic process have not been excluded.

Relatively little study has been made of the metabolic activation and the DNA adducts of fungal and plant carcinogens. The mycotoxins and hepatocarcinogens aflatoxin $B_1$ and sterigmatocystin are metabolized to highly reactive epoxides that form adducts primarily by reaction at the N-7 position of guanine residues in liver DNA (Wogan *et al.*, 1979). Likewise, the streptomyces metabolite and pancreatic carcinogen L-azaserine is metabolized to release diazoacetic acid residues that form N-7 carboxymethyl adducts of guanine bases in DNA (Zurlo *et al.*, 1982). The plant hepatocarcinogen cycasin is metabolized to a reactive species that methylates tissue DNA (Magee *et al.*, 1976). To extend this information we have recently studied other naturally occurring carcinogens from living systems. One is the fermentation product ethyl carbamate that induces tumours at several tissue sites in rodents. The others are the hepatocarcinogens safrole and estragole. These two carcinogens are plant products and belong to the group of alkenylbenzene spice flavouring agents.

*Ethyl carbamate* (*urethan*)

This simple structure (Figure 1) was discovered as a synthetic carcinogen 40 years ago in the induction of lung adenomas in mice which received it as an anaesthetic (Mirvish, 1968). Previously, ethyl carbamate had been used in some cases as a sedative in humans. Recently, ethyl carbamate was found to occur in fermented foods such as bread, yogurt, beer, and wine at levels of 1–6 $\mu g\,kg^{-1}$ (Ough, 1976). It is formed in ethanolic fermentations by the ethanolysis of carbamyl phosphate. The risks to humans of daily intakes of a few micrograms of ethyl carbamate from food sources for several decades are not known but appear to be very low. Much greater exposures of humans to ethyl carbamate occurred in Japan from approximately 1950–1975 from the use of this amide as a co-solvent for barbiturate drugs (Nomura, 1975). It is not clear whether or not it is possible to analyze the long term consequences by an epidemiological survey.

Ethyl carbamate is a versatile carcinogen (Mirvish, 1968) and can induce lung adenomas, hepatomas, mammary carcinomas, thymic lymphomas, and haemangiomas in mice. It also acts as a pure initiator of tumour formation in mouse skin and as a carcinogen in several tissues in rats and hamsters (Mirvish, 1968).

Small structural changes in ethyl carbamate generally produce large decreases in carcinogenicity (Mirvish, 1968; Shimkin *et al.*, 1969). For example, methyl carbamate is inactive and *n*-propyl and *i*-propyl carbamates have only weak activity in the mouse. Only the ethyl carbons of ethyl carbamate become bound covalently to the DNA in the mouse liver *in vivo* and methyl, *n*-propyl and *n*-butyl carbamate do not bind to liver DNA in significant amounts (Lawson & Pound, 1973). These data suggested that the ethyl group of ethyl carbamate was involved in the activation of this carcinogen *in vivo*.

A clue to an activation pathway for ethyl carbamate *in vivo* developed from the finding of the high carcinogenicity of vinyl carbamate ($CH_2{=}CH{-}O{-}CO{-}NH_2$) (Dahl *et al.*, 1978). Tests on this carbamate were prompted by the carcinogenicity of vinyl chloride in the liver and other tissues of rats and mice (IARC, 1974). Vinyl carbamate proved to be several- to many-fold more active than ethyl carbamate in a number of tissues in the mouse and rat (Dahl *et al.*, 1978, 1980); it is the first derivative of ethyl carbamate to exhibit carcinogenic activities greater than those of the parent compound. Vinyl carbamate was also mutagenic for *Salmonella typhimurium* TA 1535 and TA 100 in the presence of NADPH and mouse or rat liver mitochondrial supernatant fractions (Dahl *et al.*, 1978). This mutagenic activity was inhibited strongly by cytochome P-450 inhibitors. No mutagenic activity was observed for vinyl carbamate in the absence of added liver preparations or for ethyl carbamate in the presence or absence of liver fractions. The high carcinogenicity of vinyl carbamate and its mutagenicity with metabolic activation were consistent with our hypothesis that it might be a metabolite of ethyl carbamate and that its epoxide might be an electrophilic metabolite capable of reacting with tissue nucleophiles such as DNA. However, extensive tests by isotope dilution (Dahl *et al.*, 1978, 1980) failed to detect any conversion of ethyl carbamate to vinyl carbamate in mouse or rat liver either *in vivo* or *in vitro*. Thus, an alternative possibility is that both ethyl and vinyl carbamate are converted *in vivo* to a common reactive intermediate that effects the binding of the carbon atoms from the ethyl and vinyl groups to cellular DNA. No intermediate has been detected in our tests to date.

In view of the above results an indirect approach, based on the formation of etheno bases in hepatic RNA of vinyl chloride-treated rats (Laib & Bolt, 1977, 1978), was used to probe the metabolism of ethyl and vinyl carbamate *in vivo*. For this study hepatic RNA from mice which had received injections of [ethyl-1,2-$^3$H]- or [ethyl-1-$^{14}$C]ethyl carbamate was enzymatically hydrolyzed to the nucleoside level. The presence of 1,N$^6$-ethenoadenosine and 3,N$^4$-ethenocytidine in these hydrolysates was demonstrated by comigration on high performance liquid chromatography of $^3$H or $^{14}$C from the ethyl carbamate with synthetic standards (Ribovich *et al.*, 1982). Both the hepatic ethenoadenosine and ethenocytidine were further characterized by their conversion to acetylated products that also comigrated with the acetylated synthetic standards. Similarly, the hepatic ethenoadenosine was solvolyzed by anhydrous trifluoroacetic acid to a product that comigrated with 1,N$^6$-ethenoadenine. Figures 3 and 4 show the data obtained for ethenoadenosine with the tritiated ethyl carbamate. Similar data were obtained for 3,N$^4$-ethenocytidine with the tritiated carcinogen and for both etheno nucleosides derived from the $^{14}$C-labelled ethyl carbamate. No labelled ethenoadenosine or ethenocytidine was detected in liver RNA from mice treated with [1-$^{14}$C]ethanol, which is a metabolite of ethyl carbamate *in vivo* (Mirvish, 1968). Thus, ethyl carbamate, rather than its hydrolysis product, is required for the formation of the etheno bases in mouse liver RNA *in vivo*.

Approximately 7–10 pmol of ethenoadenosine and 2–3 pmol of ethenocytidine were found per mg of mouse liver RNA 6–12 h following the administration of 0.5 mg of ethyl carbamate per g body wt (Table II). This corresponds to ~2 and 0.7 ethenoadenosine and ethenocytidine residues, respectively, per 10$^6$ bases in RNA. Dr. Li in our laboratory has developed a method which depends on the very intense fluorescence of 1,N$^6$-ethenoadenosine in ultraviolet light (Barrio *et al.*, 1972) and permits comparisons of the formation of

**Table II** Hepatic RNA adducts formed from ethyl and vinyl carbamate in B6C3F$_1$ 12-day male mice[a]

| Carbamate | Analytical method | Dose ($\mu g\,g^{-1}$) | *pmol mg$^{-1}$ RNA* Ethenoadenosine | Ethenocytidine |
|---|---|---|---|---|
| Ethyl | [Ethyl-1,2-$^3$H or ethyl-1-$^{14}$C]ethyl carbamate | 500 | 6–10 | 2–3 |
| | Fluorometric | 500 | 5–8 | |
| Vinyl | Fluorometric | 25 | 25–30 | |
| | Fluorometric | 50 | 45–50 | |

[a]Mice killed 6–12 h after i.p. injection.

**Figure 3** High performance liquid chromatographic separation of tritiated ethenoadenosine from an enzymatic hydrolysate of hepatic RNA from adult male mice given [ethyl-1,2-$^3$H]ethyl carbamate (Ribovich *et al.*, 1982). The insert shows the pattern for a similar hydrolysate without addition of marker ethenoadenosine. A reverse-phase column was used.

**Figure 4** Further characterization of the tritiated product from hepatic RNA that comigrated with 1,N$^6$-ethenoadenosine in Figure 3. Panel A: Comigration on a cation exchange column; Panel B: Comigration on the same column after the tritiated product was partially solvolyzed with trifluoroacetic acid; Panel C: Comigration on a reverse-phase column of the tritiated product and synthetic ethenoadenosine after acetylation with acetic anhydride.

ethenoadenosine from unlabelled ethyl and vinyl carbamates. By employing a highly sensitive fluorescence detector and two successive chromatographies to isolate pure ethenoadenosine, as little as 1 pmol of ethenoadenosine can be detected per mg RNA. With this fluorometric method the yields of ethenoadenosine in hepatic rRNA from mice treated with ethyl carbamate are in good accord with those obtained with the labeled ethyl carbamate (Table II). Furthermore, administration of 25 or 50 $\mu$g vinyl carbamate per g body wt yielded several times more ethenoadenosine in the RNA than did much larger doses of ethyl carbamate (Table II). This latter result is consistent with the much greater carcinogenicity of vinyl carbamate as compared to that of ethyl carbamate and with the formation of vinyl carbamate or a derivative thereof as an important intermediate in the metabolism of ethyl carbamate to a reactive metabolite that is a precursor of the etheno adducts.

Some further data obtained with the fluorometric method for ethenoadenosine are of interest. Methyl carbamate, which is not carcinogenic and does not bind to mouse liver DNA *in vivo*, did not yield detectable amounts of ethenoadenosine in hepatic RNA. Presumably, at least a two carbon fragment is required for the formation of the etheno bases *in vivo*. The specificity for an ethyl group in these reactions *in vivo* may be quite high. It is of further interest to note the ethenoadenosine levels in non-hepatic tissues in mice administered vinyl carbamate. Vinyl carbamate is carcinogenic in the lungs and thymus of the mouse, and it gave rise to levels of ethenoadenosine in the RNA of these tissues that were similar to those produced by this carbamate in the liver. Under the same conditions rRNA from the kidney, a non-target tissue, had about one-fourth the amount of ethenoadenosine found in hepatic rRNA.

Although less information is available on the DNA adduct(s) formed *in vivo* from ethyl and vinyl carbamate, it is evident that the pattern follows that found for vinyl chloride (Laib & Bolt, 1977, 1978; Osterman-Golkar *et al.*, 1977; Laib *et al.*, 1981). Thus, following administration of ethyl or vinyl carbamate 7-(2-oxoethyl)guanine adducts, rather than the etheno bases, are formed in liver DNA. In preliminary communications Scherer *et al.* (1980) and Scherer and Emmelot (1982) reported that enzymatic hydrolysates of hepatic DNA from rats and mice given [ethyl-1-$^{14}$C]ethyl carbamate contained most of the $^{14}$C in one chromatographic peak. Neutral heating or acid hydrolysis yielded a product that comigrated with 7-(2-oxoethyl)guanine, which was further characterized by its reduction to a derivative that comigrated with 7-(2-hydroxyethyl)guanine. The ease of reduction of 7-(2-oxoethylguanine) provided a basis for a sensitive method of quantitation—i.e., by incorporation of tritium on reduction with tritiated sodium borohydride. This procedure also permits the use of unlabelled carbamates for *in vivo* studies. In recent similar studies by Dr. Li in our laboratory vinyl carbamate produced at least 20-fold higher levels of 7-(2-oxoethylguanine) in hepatic DNA of mice than were obtained from ethyl carbamate. Furthermore, for a given dose of ethyl or vinyl carbamate, the levels of this adduct in hepatic DNA were $\sim$40-fold higher than the levels of ethenoadenosine in the hepatic RNA.

The present knowledge of the metabolism of ethyl and vinyl carbamates in mouse liver is summarized in Figure 5. It is a matter for further study whether vinyl carbamate is a direct metabolite of ethyl carbamate or whether each carbamate is metabolized to a common reactive precursor of the adducts found in the hepatic nucleic acids. As shown in Figure 5, Scherer *et al.* (1981) have hypothesized that the 7-(2-oxoethyl)guanine residue in DNA may isomerize to yield a $0^6$,7-(1'-hydroxyethano)guanine adduct which might be expected to be more mutagenic than the N-7 adduct. No evidence for this derivative has been reported. However, Loeb and his colleagues (Schaaper *et al.*, 1982) have provided evidence for the mutagenicity of depurinated sites in DNA produced by carcinogenic electrophiles. This consequence may follow the formation of 7-(2-oxoethyl)guanine residues in DNA *in vivo*. In any event, further work is necessary to establish the importance of the 7-(2-oxoethyl)guanine adduct in DNA to the carcinogenic processes induced by ethyl and vinyl carbamate.

### *Safrole, estragole, and other alkenylbenzenes*

About 30 naturally occurring alkenylbenzene derivatives, usually relatively simple allyl- or propenylbenzenes with methoxy and/or methylenedioxy ring substituents, have been found as components of many plants or their essential oils (Miller & Miller, 1979; Miller *et al.*, 1983). These compounds occur in a variety of foods, but they are especially prominent as active components of many spices. Approximately 20 years ago safrole (1-allyl-3,4-methylenedioxybenzene) was found to be hepatocarcinogenic for rats and mice (IARC, 1976). Three other hepatocarcinogens have since been identified among this relatively new class of naturally occurring carcinogens, e.g., estragole (1-allyl-4-methoxybenzene), isosafrole (1-propenyl-3,4-methylenedioxybenzene), and methyleugenol (1-allyl-3,4-dimethoxybenzene) (Miller *et al.*, 1983). In addition, $\beta$-asarone (*cis*-1-propenyl-2,4,5-tri-

**Figure 5** A possible pathway of metabolism of ethyl and vinyl carbamates in mouse liver *in vivo* which leads to the formation of etheno adducts of adenosine and cytidine in RNA and a 7-(2-oxoethyl)guanine adduct in DNA. The failure to detect vinyl carbamate as a metabolite of ethyl carbamate *in vivo* or *in vitro* (Dahl *et al.*, 1978, 1980) suggests that alternative pathways to these adducts may exist.

methoxybenzene) has induced mesenchymal tumours of the small intestine in the rat (Miller *et al.*, 1983). Six related compounds did not show carcinogenic activity in a recent study (Miller *et al.*, 1983); these were anethole (*trans*-1-propenyl-4-methoxybenzene), eugenol (1-allyl-3-methoxy-4-hydroxybenzene), elemicin (1-allyl-3,4,5-trimethoxybenzene), myristicin (1-allyl-3-methoxy-4,5-methylenedioxybenzene), dill apiol (1-allyl-2,3-dimethoxy-4,5-methylenedioxybenzene), and parsley apiol (1-allyl-2,5-dimethoxy-3,4-methylenedioxybenzene). Of the derivatives shown to be carcinogenic, isosafrole and $\beta$-asarone occur only rarely in plants, while safrole, estragole, and methyleugenol occur more widely as plant constituents (Leung, 1980). Although these compounds induce high incidences of tumours under appropriate conditions, they are orders of magnitude less active than carcinogens such as the unsaturated pyrrolizidine alkaloids or aflatoxin $B_1$. Because of this fact and because they occur in the total food intake of humans as components of foods or food additives (spices) at no more than low parts $10^{-6}$ levels, the naturally occurring alkenylbenzenes appear to make a relatively small contribution to the burden of exogenous carcinogenic agents to which humans are exposed.

Safrole is a major component of oil of sassafras and a minor constituent of other essential oils, such as the oils of sweet basil and cinnamon. Estragole is a principal constituent of oil of tarragon and is found in lesser amounts in oils of sweet basil and anise and in other essential oils (Leung, 1980). The carcinogenicity and metabolism of these two carcinogens are very similar (Miller *et al.*, 1982; Miller *et al.*, 1983). Both are complete hepatocarcinogens on long-term feeding of relatively high doses in adult mice. With small doses administered only during the preweaning · period these compounds are initiators of hepatocarcinogenesis in male mice. In this situation some aspect of the normal adult male environment apparently promotes the appearance approximately one year later of gross liver tumours.

In approaching the metabolic activation of these carcinogens, note was taken of their allyl substituents, the known metabolism of ethylbenzene to 1′-hydroxyethylbenzene, and the data on the metabolism of the carcinogenic pyrrolizidine alkaloids ro form electrophilic allylic esters (references in Miller & Miller, 1979). Studies with rats and mice showed considerable metabolism of safrole and estragole to urinary glucuronides of their 1′-hydroxy metabolites (Borchert *et al.*, 1973*b*; Drinkwater *et al.*, 1976). These 1′-hydroxy metabolites were considerably more hepatocarcinogenic than the parent alkenylbenzenes (Borchert *et al.*, 1973*a*; Drinkwater *et al.*, 1976); thus these metabolites were proximate carcinogens. Evidence for the metabolism of the 1′-hydroxy metabolites to reactive derivatives *in vivo* was provided by the formation of covalently bound adducts in liver DNA, RNA, and protein (Wislocki *et al.*, 1976; Phillips *et al.*, 1981*a,b*).

The nucleophilic 1′-hydroxy metabolites, which are not themselves reactive with tissue nucleophiles, presented several possibilities for metabolism *in vivo* to electrophilic products (Figure 6). One candidate metabolite was a benzylic allylic ester. The synthetic ester 1′-acetoxysafrole was electrophilic toward nucleophiles such as guanosine and methionine (Borchert *et al.*, 1973*b*), but no evidence was found for its formation by rat liver preparations from 1′-hydroxysafrole and acetyl-coenzyme A (Wislocki *et al.*, 1976). However, 3′-phosphoadenosine-5′-phosphosulfate-dependent binding of labelled 1′-hydroxysafrole to RNA, presumably via a reactive sulfuric acid ester, was demonstrated with liver cytosols (Wislocki *et al.*, 1976). A second proposed route of activation was via metabolic epoxidation of the allylic bond. Epoxidation of safrole and estragole and of their 1′-hydroxy metabolites was catalyzed by a cytochrome P-450-dependent system in liver microsomes (Wislocki *et al.*, 1976; Swanson

**Figure 6** Some possible pathways of metabolic activation *in vivo* of safrole and estragole to form carcinogenic electrophiles.

*et al.*, 1981), and, as expected, these epoxides were electrophilic. 1′-Oxosafrole was also investigated, since previous work (Oswald *et al.*, 1971) suggested its formation *in vivo* in the rat from safrole. This derivative was electrophilic toward guanosine, presumably through addition of the amino group of this nucleophile to the allylic double bond (Wislocki *et al.*, 1976).

The mutagenicities of these electrophiles were determined as another measure of their electrophilicity in a biological situation. The 1′-acetoxy and 2′,3′-oxide derivatives of safrole, estragole, and their 1′-hydroxy metabolites were mutagenic for *Salmonella typhimurium* TA100, but the 1′-oxo derivatives of safrole and estragole were inactive (Wislocki *et al.*, 1977; Swanson *et al.*, 1979; Phillips *et al.*, 1981*a*). The carcinogenicities of many of the known and possible electrophilic metabolites of safrole and estragole have been determined in rats and/or mice (Borchert *et al.*, 1973*a*; Wislocki *et al.*, 1977; Miller *et al.*, 1983). Although some of the acetoxy and epoxy derivatives showed carcinogenic activity, especially at sites of application, these activities were generally lower than those of 1′-hydroxysafrole and 1′-hydroxyestragole; possibly little of these reactive compounds reached critical sites in the cells. 1′-Oxosafrole showed no carcinogenic activity in these tests.

In order to obtain more direct information on the identities of the electrophilic metabolites that might be of importance in carcinogenesis by estragole, comparisons were made of the adducts formed *in vitro* by reaction of deoxynucleosides with electrophiles derived from 1′-hydroxyestragole and nucleoside adducts derived by enzymatic hydrolyses of hepatic DNA from mice injected with 1′-[2′,3′-$^3$H]hydroxyestragole. The adducts formed *in vivo* were cochromatographed by high performance liquid chromatography with nucleosides derived from the reaction of 1′-acetoxyestragole, 1′-hydroxyestragole-2′,3′-oxide, or 1′-oxoestragole with [$^{14}$C]deoxyguanosine or [$^{14}$C]deoxyadenosine. The four nucleoside adducts (I–IV) obtained from the hepatic DNA of mice injected with the tritiated 1′-hydroxyestragole did not comigrate with any major reaction products of either 1′-hydroxyestragole-2′,3′-oxide or 1′-oxoestragole with deoxyguanosine or deoxyadenosine. However, the *in vivo* adducts I–III comigrated with 3 adducts from the reaction of 1′-acetoxyestragole with deoxyguanosine and adduct IV comigrated with the reaction product of the acetoxy derivative with deoxyadenosine. On the basis of these chromatographic data it was concluded that the 4 principal adducts formed *in vivo* in the hepatic DNA from 1′-hydroxyestragole were products of the reaction of some metabolically-formed ester of 1′-hydroxyestragole with deoxyguanosine and deoxyadenosine residues in the DNA. Large scale preparation of the 4 adducts permitted the characterization of their structures (Phillips *et al.*, 1981*a*) (Figure 7). Two of the adducts (II and III) consist of 3′-isoestragole residues bonded in the *trans-* or *cis-*configurations to the 2-amino group of deoxyguanosine. A third adduct (I) consists of a 1′-estragole residue attached to the 2-amino group of deoxyguanosine. The fourth adduct (IV) is comprised of a 3′-isoestragole residue attached to the 6-amino group of deoxyadenosine. Similar procedures were used to demonstrate that the adducts formed in mouse liver DNA *in vivo* from 1′-hydroxysafrole have structures that are entirely analogous to those derived from 1′-hydroxyestragole (Phillips *et al.*, 1981*b*).

These data suggest strongly that esters of 1′-hydroxysafrole and 1′-hydroxyestragole are the major electrophilic metabolites that react with the hepatic DNA of mice treated with these two carcinogens (Figure 8). As noted above Wislocki *et al.* (1976) had obtained evidence for the formation of a sulfuric acid ester of 1′-hydroxysafrole by a 3′-phosphoadenosine-5′-phosphosulfate-dependent sulfotransferase activity in mouse and rat liver cytosols. Recently, Eric Boberg in our laboratory has investigated the role of this ester in hepatocarcinogenesis by 1′-hydroxysafrole in the mouse. Using an assay for the liver cytosolic sulfotransferase activity toward 1′-hydroxysafrole adapted from that of Wislocki *et al.* (1976), he showed that hepatic sulfotransferase activity is detectable on the day of birth of C57BL/6 x C3H/He (B6C3F$_1$) mice and that it increases up to 3 weeks of age; thereafter the level decreases and by day 60 the activity is in the same range as on the day of birth. Pentachlorophenol, which has been widely used as an inhibitor of sulfotransferase activity (Meerman *et al.*, 1980, 1981), inhibited the sulfotransferase activity for 1′-hydroxysafrole in male and female mouse liver at a 10 $\mu$molar level. Likewise, as shown in Table III, the administration of 0.05% of pentachlorophenol in the diet of adult female CD-1 mice strongly inhibits the formation of DNA, RNA, and protein-bound adducts formed after the injection of a single dose of tritiated 1′-hydroxysafrole to ~10–25% of those in the liver of the control mice. Furthermore, when 1′-hydroxysafrole was fed in the diet of adult CD-1 mice, hepatic tumor formation was greatly inhibited if the mice were fed simultaneously 0.05% of pentachlorophenol (Table IV). Thus, pentachlorophenol inhibits the hepatic sulfotransferase that catalyzes the formation of the sulfuric acid ester of 1′-hydroxysafrole, reduces dramatically the levels of DNA, RNA, and protein adducts of this proximate carcinogen in the livers of mice treated with this sulfotransferase inhibitor, and

**Figure 7** Structures of the major DNA adducts formed in mouse liver *in vivo* from 1′-hydroxyestragole (Phillips *et al.*, 1981*a*).

also inhibits hepatic tumour formation by 1′-hydroxysafrole. These data strongly implicate the sulfuric acid ester as a critical metabolite in the hepatic carcinogenicity of 1′-hydroxysafrole.

**Table III** The inhibition of the covalent binding of [2′,3′-$^3$H]1′-hydroxysafrole to hepatic macromolecules of CD-1 adult female mice

The mice were fed the indicated diets for 4 weeks before administration of a single 3-mg dose of [2′,3′-$^3$H]1′-hydroxysafrole by stomach tube. They were killed 5 h later for isolation of the hepatic macromolecules.

| | *Hepatic adducts (pmol mg$^{-1}$ of macromolecule)* | | |
|---|---|---|---|
| *Diet* | *DNA* | *RNA* | *Protein* |
| Control | 125±15[a] | 420±95 | 365±90 |
| 0.05% Pentachlorophenol | 20±10 | 45±25 | 100± 5 |

[a]Each value is the average ± the standard deviation for 3 pools of liver (2 livers per pool).

**Table IV** The inhibition by pentachlorophenol of the induction of hepatomas in CD-1 adult female mice fed 1′-hydroxysafrole

Groups of 36 adult female CD-1 mice were fed diets containing 1′-hydroxysafrole for 12 months. The experiment was terminated at 16 months.

| *Dietary 1′-hydroxysafrole (%)* | *Dietary pentachloro-phenol (%)* | *Mice with hepatomas (%)*[a] | *Average number of hepatomas per mouse* |
|---|---|---|---|
| 0.27 | 0 | 81 | 1.6 |
| 0.27 | 0.05 | 17 | 0.2 |
| 0.14 | 0 | 50 | 1.1 |
| 0.14 | 0.05 | 6 | 0.06 |
| 0 | 0 | 0 | 0.0 |
| 0 | 0.05 | 7 | 0.07 |

[a]The data are based on the number of mice that survived for at least 12 months, when the first mice died with gross tumours.

**Figure 8** The proposed pathway of metabolic activation of safrole and estragole in mouse liver to form DNA adducts.

Further evidence consistent with this conclusion was obtained by the use of mice with the brachymorphic trait. Sugahara and Schwartz (1979, 1982) found that brachymorphic mice, which are characterized by an undersulfation of cartilage glycosaminoglycans, have a low capacity for the formation of 3′-phosphoadenosine-5′-phosphosulfate in the liver and some other tissues as compared to normal mice. Dr. Alan Poland at McArdle recognized that brachymorphic mice might be useful tools in the analysis of the roles of sulfate esters of certain carcinogens in the carcinogenic processes that they induce since the livers of these mice have a reduced capacity for the formation of the sulfate ester of *p*-nitrophenol (Lyman & Poland, 1983). Accordingly, in collaboration with Dr. Poland we have bred $B6C3F_2$ mice from stock which carries the brachymorphic trait. Among the littermates there are ~25% that are homozygous recessives for this trait and are recognizable by their characteristic short tails, short legs, and dome-shaped heads. The phenotypically normal littermates include heterozygous mice that contain both a normal and a brachymorphic gene and homozygous mice that contain 2 normal genes at the brachymorphic locus. These two genotypes cannot be distinguished phenotypically. As shown in Table V the livers of 12-day-old brachymorphic mice contain only ~20% as much of the DNA and RNA adducts of tritiated

**Table V** Hepatic adducts in 12-day-old male $B6C3F_2$ mice treated with [2′,3′-$^3$H]1′-hydroxysafrole

The mice were injected intraperitoneally with a trioctanoin solution of [2′,3′-$^3$H]1′-hydroxysafrole (0.20 μmol g$^{-1}$ body wt) and were killed 9 h later for isolation of the hepatic macromolecules.

| | *Hepatic adducts (pmol mg$^{-1}$ of macromolecule)* | | |
|---|---|---|---|
| *Phenotype* | *DNA* | *RNA* | *Protein* |
| Normal | 110±17[a] | 200±34 | 210±33 |
| Brachymorphic[b] | 16± 1 | 19± 1 | 57± 2 |

[a]Each analysis is the average ± the standard deviation for 3 pools of 5–6 livers each.

[b]These mice are deficient in the hepatic synthesis of 3′-phosphoadenosine 5′-phosphosulfate.

1'-hydroxysafrole as did their phenotypically normal littermates after the administration of this proximate carcinogen. When similar mice were initiated for hepatocarcinogenesis by the injection of 1'-hydroxysafrole only during the first 3 weeks of life and then maintained without further treatment for up to 15 months of age, the brachymorphic mice had only ~20% of the hepatic tumour incidence of the phenotypically normal mice (Table VI). The deficiency in hepatic 3'-phosphadenosine-5'-phosphosulfate, reduced formation of hepatic nucleic acid adducts of 1'-hydroxysafrole, and reduced initiation of tumour formation in the liver by this proximate carcinogen are strongly correlated in these mice. Thus, all of the data in Tables III–VI are consistent with the concept that the sulfuric acid ester of 1'-hydroxysafrole is a major ultimate carcinogenic metabolite of this proximate carcinogen. Because of the close similarity of the metabolism of estragole to that of safrole, we presume that the sulfuric acid ester of 1'-hydroxyestragole is similarly important in hepatocarcinogenesis by estragole and its 1'-hydroxy metabolite. However, we do not yet have data confirming this presumption.

**Table VI** The incidences of hepatomas in normal and brachymorphic male B6C3F$_2$ mice treated with 1'-hydroxysafrole at 1–22 days of age

The mice were injected on Days 1, 8, 15, and 22 after birth with trioctanoin solutions of 1'-hydroxysafrole; the relative amounts injected on these days were in the ratio of 1:2:4:8. The experiment was terminated at 15 months.

| *1'-Hydroxy-safrole (μmol/mouse)* | *Phenotype* | *Mice with hepatomas (%)* | *Average number of hepatomas per mouse* |
|---|---|---|---|
| 3.7 | Nornal | 71[a] | 2.2 |
| 3.7 | Brachymorphic[b] | 9 | 0.2 |
| 1.8 | Normal | 50 | 1.3 |
| 1.8 | Brachymorphic | 10 | 0.1 |
| 0 | Normal | 14 | 0.1 |
| 0 | Brachymorphic | 2 | 0.02 |

[a]The data are based on the number of mice that survived for at least 12 months, when the first mice died with gross tumours.

[b]These mice are deficient in the synthesis of 3'-phospho-adenosine 5'-phosphosulfate.

While the genesis and nature of the major adducts in hepatic DNA derived from safrole and estragole are reasonably well defined, the important problem remains of establishing the role of these adducts in the initiation of carcinogenesis in the mouse liver by these agents. Likewise, the possible roles of the macromolecular adducts in the promotion stage require further investigation.

*Perspectives*

As noted early in this lecture, it seems likely that more carcinogenic agents exist in the living world than are now known. A primary basis for this statement is that, although only a relatively small number of cellular components have been tested for carcinogenic activity, over 30 of these diverse structures have been found to be carcinogenic. Man's food is derived almost entirely from living systems and constitutes by far the major daily source of non-nutritive chemicals, which may include compounds that could increase (carcinogens, initiators, promoters, cocarcinogens, etc.) or decrease (anti-carcinogens, anti-mutagens, etc.) the incidence of cancers in humans. The primary evidence that diet may play a role in the occurrence of major human cancers comes from epidemiological studies on the incidences of cancers among populations in different countries and migrants between them, as well as investigations on dietary differences between population groups (Hirayama, 1979; Doll & Peto, 1981; National Research Council, 1982). While some evidence points to a causal role of certain naturally occurring dietary agents (e.g., aflatoxins in hepatic cancer) definitive evidence implicating dietary components in the aetiologies of human cancers is generally lacking. This situation probably reflects the multi-factorial nature of carcinogenesis and anti-carcinogenesis in man. Thus, in addition to the strong associations of tobacco, alcoholic drinks, and ultraviolet light with the genesis of specific cancers, the probability of formation of cancers in the last third of the life-span in a human being may reflect in part long-term dietary intakes of agents that increase cancer formation and/or of agents that inhibit this process. Extensive experimental studies have revealed a wide variety of mutagenic agents (i.e., possible initiators of carcinogenesis) in natural and over-heated foods (Sugimura *et al.*, 1982; Stich, 1982). Similarly, at least a dozen naturally occurring substances of varied structure appear to be anti-carcinogenic (Wattenberg, 1979). Further innovative research is needed on these interactions, especially in studies with human beings.

Several naturally occurring components of living systems are highly potent carcinogens in experimental animals. These include aflatoxin $B_1$, cycasin, the unsaturated pyrrolizidine alkaloids, and the as yet uncharacterized carcinogen(s) in bracken fern (Miller & Miller, 1979). Ethyl carbamate and the carcinogenic alkenylbenzenes have exhibited moderate-to-weak carcinogenic activity in experimental animals. The carcinogenic potentials of none of the naturally occurring carcinogens from living systems in human beings are known. Since species and strain differences can play important roles in the activities of chemical carcinogens (Langenbach *et al.*, 1983), it is possible that even low life-time intakes of so-called "weak" carcinogens could induce cancers in some humans with especially predisposing genetic backgrounds and dietary habits.

The molecular nature of carcinogenic processes induced by chemical carcinogens is still unclear. Extensive progress has been made on the metabolism of chemical carcinogens to reactive electrophiles *in vivo* and on the structural characterization and properties of adducts of these metabolites with DNA and other informational macromolecules. However, these hypotheses have generally lacked heuristic value for the prediction of specific molecular mechanisms that could be tested. Thus, research on chemical carcinogenesis has reached an apparent impasse in attempts to extend the data on the formation of specific DNA adducts from electrophilic metabolites to the determination of the molecular lesions directly responsible for the initiation of carcinogenesis. Recent observations on the occurrence in normal and tumour tissues from experimental animals and humans of DNA sequences homologous to portions of the transforming genes of oncogenic retroviruses and on the transforming activity of these sequences for certain cell lines (Bishop, 1982; Rigby, 1982; Weiss, 1982; Weinberg, 1982; Newmark, 1983) have provided new ideas on the possible nature of the carcinogen-induced lesions that may be involved in malignant transformation by chemicals. Exploration of this phenomenon, as well as continuing analyses of other effects of electrophilic reactions of the macromolecules in relation to the carcinogen-induced transformation, should bring us much closer to a molecular understanding of malignant transformation.

It is a pleasure to acknowledge the collaboration of Eric Boberg and Drs. Peter Borchert, Gary A. Dahl, Norman R. Drinkwater, Yaguan Li, David H. Phillips, Martin L. Ribovich, Anne B. Swanson, and Peter G. Wislocki in these studies during the past decade. The work in the authors' laboratory was supported by Grants CA-07175, CA-09135, CA-09020, and CA-22484 from the National Cancer Institute, United States Public Health Service.

### References

BARRIO, J.R., SECRIST, J.A., III & LEONARD, N.J. (1972). Fluorescent adenosine and cytidine derivatives. *Biochem. Biophys. Res. Commun.*, **46**, 597.

BISHOP, J.M. (1982). Oncogenes. *Scientific American*, **246**, 80.

BORCHERT, P., MILLER, J.A., MILLER, E.C. & SHIRES, T.K. (1973*a*). 1′-Hydroxysafrole, a proximate carcinogenic metabolite of safrole in the rat and mouse. *Cancer Res.*, **33**, 590.

BORCHERT, P., WISLOCKI, P.G., MILLER, J.A. & MILLER, E.C. (1973*b*). The metabolism of the naturally occurring hepatocarcinogen safrole to 1′-hydroxysafrole and the electrophilic reactivity of 1′-acetoxysafrole. *Cancer Res.*, **33**, 575.

DAHL, G.A., MILLER, E.C. & MILLER, J.A. (1980). Comparative carcinogenicities and mutagenicities of vinyl carbamate, ethyl carbamate, and ethyl N-hydroxycarbamate. *Cancer Res.*, **40**, 1194.

DAHL, G.A., MILLER, J.A. & MILLER, E.C. (1978). Vinyl carbamate as a promutagen and a more carcinogenic analog of ethyl carbamate. *Cancer Res.*, **38**, 3793.

DOLL, R. & PETO, R. (1981). The causes of cancer: Quantitative estimates of avoidable risks of cancer in the U.S. today. *J. Natl Cancer Inst.*, **66**, 1192.

DRINKWATER, N.R., MILLER, E.C., MILLER, J.A. & PITOT, H.C. (1976). The hepatocarcinogenicity of estragole (1-allyl-4-methoxybenzene) and 1′-hydroxyestragole in the mouse and the mutagenicity of 1′-acetoxyestragole in bacteria. *J. Natl Cancer Inst.*, **57**, 1323.

HIRAYAMA, T. (1979). Epidemiological evaluation of the role of naturally occurring carcinogens and modulators of carcinogenesis. In *Naturally Occurring Carcinogens-Mutagens and Modulators of Carcinogenesis*, p. 359. (Eds. E.C. Miller, J.A. Miller, I. Hirono, T. Sugimura, & S. Takayama). Tokyo: Japan Scientific Societies Press. Baltimore: University Park Press.

*IARC Monographs on the Evaluation of Carcinogenic Risk of Chemicals to Man, Safrole, Isosafrole, and Dihydrosafrole*, Vol. 10, p. 231 (1976). Lyon, France: International Agency for Research on Cancer.

*IARC Monographs on the Evaluation of Carcinogenic Risk of Chemicals to Man, Vinyl Chloride*, Vol. 7, p. 291. (1974). Lyon, France: International Agency for Research on Cancer.

LAIB, R.J. & BOLT, H.M. (1977). Alkylation of RNA by vinyl chloride metabolites *in vivo*: Formation of 1,$N^6$-ethenoadenosine. *Toxicology*, **8**, 185.

LAIB, R.J. & BOLT, H.M. (1978). Formation of 3,$N^4$-ethenocytidine moieties in RNA by vinyl chloride metabolites *in vitro* and *in vivo*. *Arch. Toxicol.*, **39**, 235.

LAIB, R.J., GWINNER, L.M. & BOLT, H.M. (1981). DNA alkylation by vinyl chloride metabolites: Etheno derivatives or 7-alkylation of guanine? *Chem.-Biol. Interactions*, **37**, 219.

LANGENBACH, R., NESNOW, S. & RICE, J.M. (eds.) (1983). *Organ and Species Specificity in Chemical Carcinogenesis*, Basic Life Sciences, vol. 24. New York: Plenum Press.

LAWSON, T.A. & POUND, A.W. (1973). The interaction of carbon-14-labelled alkyl carbamates, labelled in the alkyl and carbonyl positions, with DNA *in vivo*. *Chem.-Biol. Interactions*, **6,** 99.

LEUNG, A.Y. (1980). *Encyclopedia of Common Natural Ingredients Used in Food, Drugs, and Cosmetics*, New York: John Wiley & Sons.

LYMAN, S.D. & POLAND, A. (1983). Effect of the brachymorphic trait in mice on xenobiotic sulfate ester formation. *Biochem. Pharmacol.* (in press).

MAGEE, P.N., MONTESANO, R. & PREUSSMANN, R. (1976). *N*-Nitroso compounds and related carcinogens. In *Chemical Carcinogens, ACS Monograph 173*, p. 491. (Ed. C.E. Searle). Washington: American Chemical Society.

MEERMAN, J.H., BELAND, F.A. & MULDER, G.J. (1981). Role of sulfate in the formation of DNA adducts from N-hydroxy-2-acetylaminofluorene in rat liver *in vivo*. Inhibition of N-acetylated aminofluorene adduct formation by pentachlorophenol. *Carcinogenesis*, **2,** 413.

MEERMAN, J.H., VAN DOORN, A.B.D. & MULDER, G.D. (1980). Inhibition of sulfate conjugation of *N*-hydroxy-2-acetylaminofluorene in isolated perfused rat liver and in the rat *in vivo* by pentachlorophenol and low sulfate. *Cancer Res.*, **40,** 3772.

MILLER, E.C. & MILLER, J.A. (1979). Naturally occurring chemical carcinogens that may be present in foods. In *International Review of Biochemistry, Biochemistry of Nutrition 1A*, p. 123. (Eds. A. Neuberger & T.J. Jukes). Baltimore: University Park Press.

MILLER, E.C. & MILLER, J.A. (1981). Searches for ultimate chemical carcinogens and their reactions with cellular macromolecules. *Cancer*, **47,** 2327.

MILLER, E.C., SWANSON, A.B., PHILLIPS, D.H., FLETCHER, T.L., LIEM, A. & MILLER, J.A. (1983). Structure-activity studies of the carcinogenicities in the mouse and rat of some naturally occurring and synthetic alkenylbenzene derivatives related to safrole and estragole. *Cancer Res.*, **43,** 1124.

MILLER, J.A., MILLER, E.C. & PHILLIPS, D.H. (1982). The metabolic activation and carcinogenicity of alkenylbenzenes that occur naturally in many spices. In *Carcinogens and Mutagens in the Environment*, Vol. 1, *Food Products*, p. 83. Ed. H.F. Stich. Boca Raton, Florida: CRC Press.

MIRVISH, S. (1968). The carcinogenic action and metabolism of urethan and N-hydroxyurethan. *Adv. Cancer Res.*, **11,** 1.

NATIONAL RESEARCH COUNCIL (1982). *Diet, Nutrition, and Cancer*. Washington, D.C.: National Academy Press.

NEWMARK, P. (1983). Chromosome translocations. Still more about oncogenes. *Nature*, **301,** 111.

NOMURA, T. (1975). Urethan (ethyl carbamate) as a cosolvent of drugs commonly used parenterally in humans. *Cancer Res.*, **35,** 2895.

OSTERMAN-GOLKAR, S., HULTMARK, D., SEGERBÄCK, D. & 4 others. (1977). Alkylation of DNA and proteins in mice exposed to vinyl chloride. *Biochem. Biophys. Res. Commun.*, **76,** 259.

OSWALD, E.O., FISHBEIN, L., CORBETT, B.J. & WALKER, M.P. (1971). Identification of tertiary aminomethylenedioxypropiophenones as urinary metabolites of safrole in the rat and guinea pig. *Biochim. Biophys. Acta*, **230,** 237.

OUGH, C.S. (1976). Ethylcarbamate in fermented beverages and foods. I. Naturally occurring ethylcarbamate. *J. Agric. Food Chem.*, **24,** 323.

PHILLIPS, D.H., MILLER, J.A., MILLER, E.C. & ADAMS, B. (1981*a*). Structures of the DNA adducts formed in mouse liver after administration of the proximate hepatocarcinogen 1′-hydroxyestragole. *Cancer Res.*, **41,** 176.

PHILLIPS, D.H., MILLER, J.A., MILLER, E.C. & ADAMS, B. (1981*b*). The $N^2$-atom of guanine and the $N^6$-atom of adenine residues as sites for covalent binding of metabolically activated 1′-hydroxysafrole to mouse-liver DNA *in vivo*. *Cancer Res*, **41,** 2664.

RIBOVICH, M.L., MILLER, J.A., MILLER, E.C. & TIMMINS, L.G. (1982). Labeled 1,$N^6$-ethenoadenosine and 3,$N^4$-ethenocytidine in hepatic RNA of mice given [ethyl-1,2-$^3$H or ethyl-1-$^{14}$C]ethyl carbamate (urethan). *Carcinogenesis*, **3,** 539.

RIGBY, P.W.J. (1982). The oncogenic circle closes. *Nature*, **297,** 451.

SCHAAPER, R.M., GLICKMAN, B.W. & LOEB, L.A. (1982). Role of depurination in mutagenesis by chemical carcinogens. *Cancer Res.*, **42,** 3480.

SCHERER, E. & EMMELOT, P. (1982). 7-(2-Oxoethyl)guanine, a possibly promutagenic base, in DNA modified *in vivo* by ethyl carbamate (urethane). *Abstracts*, Proc. 13th Internatl Cancer Congress.

SCHERER, E., STEWARD, A.P. & EMMELOT, P. (1980). Formation of precancerous islands in rat liver and modification of DNA by ethyl carbamate: Implications for its metabolism. In *Mechanisms of Toxicity and Hazard Evaluation*, p. 249. (Eds. B. Holmstedt, R. Lauwerys, M. Mercier, & M. Roberfroid). Amsterdam: Elsevier/North Holland Biomedical Press.

SCHERER, E., VAN DER LAKEN, C.J., GWINNER, I.M., LAIB, R.J. & EMMELOT, P. (1981). Modification of deoxyguanosine by chloroethylene oxide. *Carcinogenesis*, **2,** 671.

SHIMKIN, M.B., WIEDER, R., McDONOUGH, M., FISHBEIN, L. & SWERN, D. (1969). Lung tumor response in strain A mice as a quantitative bioassay of carcinogenic activity of some carbamates and aziridines. *Cancer Res.*, **29,** 2184.

STICH, H.F. (ed.) (1982). *Carcinogens and Mutagens in the Environment*, Vol. 1, *Food Products*, Boca Raton, Florida: CRC Press.

SUGAHARA, K. & SCHWARTZ, N.B. (1979). Defect in 3′-phosphoadenosine 5′-phosphosulfate formation in brachymorphic mice. *Proc. Natl Acad. Sci., U.S.A.*, **76,** 6615.

SUGAHARA, K. & SCHWARTZ, N.B. (1982). Defect in 3′-phosphoadenosine 5′phosphosulfate synthesis in brachymorphic mice. *Arch Biochem. Biophys.*, **214,** 602.

SUGIMURA, T., KONDO, S. & TAKEBE, H. (eds.) (1982). *Environmental Mutagens and Carcinogens*. New York: Alan R. Liss.

SWANSON, A.B., CHAMBLISS, D.D., BLOMQUIST, J.C., MILLER, E.C. & MILLER, J.A. (1979). The mutagenicities of safrole, estragole, eugenol, *trans*-anethole, and some of their known or possible metabolites for *Salmonella typhimurium* mutants. *Mutat. Res.*, **60,** 143.

SWANSON, A.B., MILLER, E.C. & MILLER, J.A. (1981). The side-chain epoxidation and hydroxylation of the hepatocarcinogens safrole and estragole and some related compounds by rat and mouse liver microsomes. *Biochim. Biophys. Acta*, **673**, 504.

WATTENBERG, L.W. (1979). Naturally occurring inhibitors of chemical carcinogenesis. In *Naturally Occurring Carcinogens—Mutagens and Modulators of Carcinogenesis*, p. 315. (Eds. E.C. Miller, J.A. Miller, I. Hirono, T. Sugimura & S. Takayama) Tokyo: Japan Scientific Societies Press, Baltimore: University Park Press.

WEINBERG, R.A. (1982). Fewer and fewer oncogenes. *Cell*, **30**, 3.

WEISS, R. (1982). The *myc* oncogene in man and birds. *Nature*, **299**, 9.

WISLOCKI, P.G., BORCHERT, P., MILLER, J.A. & MILLER, E.C. (1976). The metabolic activation of the carcinogen 1′-hydroxysafrole *in vivo* and *in vitro* and the electrophilic reactivities of possible ultimate carcinogens. *Cancer Res.*, **36**, 1686.

WISLOCKI, P.G., MILLER, E.C., MILLER, J.A., McCOY, E.C. & ROSENKRANZ, H.S. (1977). Carcinogenic and mutagenic activities of safrole, 1′-hydroxysafrole, and some known or possible metabolites. *Cancer Res.*, **37**, 1883.

WOGAN, G.N., CROY, R.G., ESSIGMANN, J.M. & 4 others. (1979). Mechanisms of action of aflatoxin $B_1$ and sterigmatocystin: Relationships of macromolecular binding to carcinogenesis. In *Environmental Carcinogenesis: Occurrence, Risk Evaluation, and Mechanisms*, p. 97. (Eds. P. Emmelot & E. Kriek). Amsterdam/New York/Oxford: Elsevier/North-Holland Biomedical Press

ZURLO, J., CURPHEY, T.J., HILEY, R. & LONGNECKER, D.S. (1982). Identification of 7-carboxymethylguanine in DNA from pancreatic acinar cells exposed to azaserine. *Cancer Res.*, **42**, 1286.